# Source Partitioning Using Stable Isotopes: Coping with Too Much Variation

**DOI:** 10.1371/journal.pone.0009672

**Published:** 2010-03-12

**Authors:** Andrew C. Parnell, Richard Inger, Stuart Bearhop, Andrew L. Jackson

**Affiliations:** 1 School of Mathematical Sciences, University College Dublin, Dublin, Ireland; 2 Centre for Ecology and Conservation, School of Biosciences, University of Exeter, Penryn, Cornwall, United Kingdom; 3 Department of Zoology, School of Natural Sciences, Trinity College Dublin, Dublin, Ireland; University of Bristol, United Kingdom

## Abstract

**Background:**

Stable isotope analysis is increasingly being utilised across broad areas of ecology and biology. Key to much of this work is the use of mixing models to estimate the proportion of sources contributing to a mixture such as in diet estimation.

**Methodology:**

By accurately reflecting natural variation and uncertainty to generate robust probability estimates of source proportions, the application of Bayesian methods to stable isotope mixing models promises to enable researchers to address an array of new questions, and approach current questions with greater insight and honesty.

**Conclusions:**

We outline a framework that builds on recently published Bayesian isotopic mixing models and present a new open source R package, SIAR. The formulation in R will allow for continued and rapid development of this core model into an all-encompassing single analysis suite for stable isotope research.

## Introduction

Stable isotope approaches are an important ecological tool, enabling increasingly sophisticated questions to be addressed in a number of fields [1], [2] although the majority of work has been in the area of animal foraging and resource partitioning [e.g. 3]. Much progress has been facilitated by isotopic mixing models which allow researchers to estimate the proportional contribution of sources (dietary items) within a mixture (consumer tissue), and thereby infer diet composition [4], [5].

Numerous approaches to solving isotopic mixing models have been proposed [6], [7], [8], [9], [10], although those developed by Phillips & co-authors have been the most widely embraced. For mathematically determined systems, simple linear mixing models [6] were used to find unique solutions, the assumption being that there is no variability within sources. An extension to these, IsoError [10], allowed variation to be propagated to produce uncertainty within the outputs, being more appropriate in natural systems. In underdetermined systems where the number of sources is greater than the number of isotopes plus one, and no exact solution exists, the standard approach has been to use IsoSource [9] using an iterative algorithm, producing a range of feasible solutions, based on an arbitrary user-defined threshold.

Although these approaches have been successful, some recurring issues remain:

1. The task of dealing with uncertainties inherent in all types of biological systems, particularly ecological situations
2. Working with underdetermined systems, where there are many more potential sources than isotopes.
3. Incorporating variability into the input parameters, such as the end members (consumers), sources and trophic enrichment factors (TEFs).
4. Dealing with external sources of variation not connected to isotopic uncertainty (such as physiological differences or unidentified minor dietary sources).

In general, some existing models can incorporate variability but are constrained by the number of sources, e.g. IsoError [10]. While later models, e.g. IsoSource [9] can cope with multiple sources, they cannot incorporate uncertainty and variation. Perhaps most frustratingly, the outputs from these models represent a range of feasible solutions, with no quantification as to which solutions are most likely. Furthermore, none of these models account for issues 3 & 4 above, particularly variation in TEFs, which can be problematic [11], [12].

Bayesian inference offers to circumvent the limitations indicated above, incorporating many more sources of variability within the model, while allowing for multiple dietary sources and then generating potential dietary solutions as true probability distributions. We present a novel methodology for analysing mixing models implemented in the software package SIAR (Stable Isotope Analysis in R) [13]. SIAR is available to download from the packages section of the Comprehensive R Archive Network site (CRAN) - http://cran.r-project.org/. SIAR is similar in many regards to MixSIR, a recently published Bayesian mixing model [14] which is providing novel insights in a variety of situations [15], [16], [17]. Aside from relatively minor differences in the fitting algorithms implemented in SIAR and MixSIR (SIAR uses MCMC while MixSIR uses Sample Importance Resampling) the two models differ fundamentally such that SIAR includes an overall residual error term lacking from MixSIR [18]. Some debate remains as to the appropriateness of including or omitting the residual error term with arguments for model simplicity favouring omission in some cases [19]. However, we maintain that unknown sources of error on the observed data, beyond that quantified by errors on the sources and the trophic enrichment factors should not be ignored from a philosophical stand-point and a residual term should always be included (as one would routinely expect in any linear model) [20].

## Methods

First, we outline the algebra for our system. We deal with a generic situation where data comprise *N* measurements on *J* isotopes with *K* sources:

*X_ij_* = observed isotope value *j* of the consumer *i*.
*s_jk_* = source value *k* on isotope *j*; normally distributed with mean *μ_jk_* and variance *ω_jk_^2^*.
*c_jk_* = TEF for isotope *j* on source *k*; normally distributed with mean *λ_jk_* and variance *τ_jk_^2^*.
*p_k_* = dietary proportion of source *k*; estimated by the model.
*q_jk_* = concentration of isotope *j* in source *k* [see 20]
*ε_ij_* = residual error, describing additional inter-observation variance not described by the model, *σ_j_^2^* estimated by the model.

The model is formulated as follows:
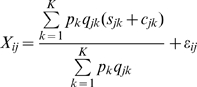
(1)

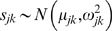
(2)

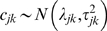
(3)

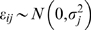
(4)A key advance is that the Bayesian paradigm allows for uncertainty in all the parameters. The two most important here are *p* and *σ^2^*, controlling proportional contribution and residual variance respectively. Model fitting is hierarchical, offering unbounded flexibility in adding complexity. Moreover, the Bayesian approach allows for external (prior) information to be incorporated, further narrowing the precision of estimated dietary proportions. This prior information may be vague or informative, e.g. corresponding to the volume of existing knowledge regarding *a priori* knowledge about the diet of an animal. A natural prior distribution for *p_k_* is the Dirichlet, a generalisation of the Beta distribution [21].

The Dirichlet distribution treats each source input as independent but requires they sum to unity. SIAR allows users to specify prior information on the mean proportions (that sum to unity) for each dietary source and a standard deviation for the first of these proportions; this is used to generate *K α* values. This external information directs the model according to the user's expert knowledge. The Dirichlet prior does not allow the user to specify individual uncertainties for each proportion, but the prior as input does match exactly what the model receives and uses it to draw consistent proportions [18].

The generated marginal distributions of a Dirichlet distribution with *K α* values [21] can be explored by defining:
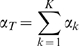
(5)and then the characteristics of the distribution are given by:
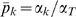
(6)

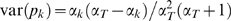
(7)

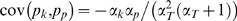
(8)where 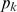 and 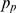 (and their associated Dirichlet parameters 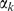 and 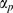) are the *k* ^th^ and *p* ^th^ dietary proportions. The default SIAR model sets each of the *α* values to 1. This corresponds to a vague prior with each source having prior mean *1/K* and prior variance *(K−1)/(K^2^(K+1))*. The Dirichlet-distributed default prior used by SIAR is designed to be vague so that the results are primarily influenced by the data. More informative prior distributions are available as part of the SIAR software. Furthermore, although the Dirichlet prior distribution has a prescribed covariance structure, the posterior distributions may have more complicated structures, yielding important information about how well the model can discriminate between sources. SIAR includes diagnostic matrix plots for exploring this covariance structure. Strong negative correlation between two posterior *p_k_* values implies that one source is simply being traded off against the other and the model is unable to isolate the contribution of either in isolation. A vague prior is also assigned to *σ*.

Model fitting is via Markov chain Monte Carlo (MCMC) which produces simulations of plausible values of *p_k_* consistent with the data. The estimated values of the parameters after taking into account the data and the prior information are known as posterior distributions, representing a true probability density for the parameters of interest. The R package SIAR allows full access to these posterior densities so that users can create any summary of the output they require.

## Results

**Figure 1:**
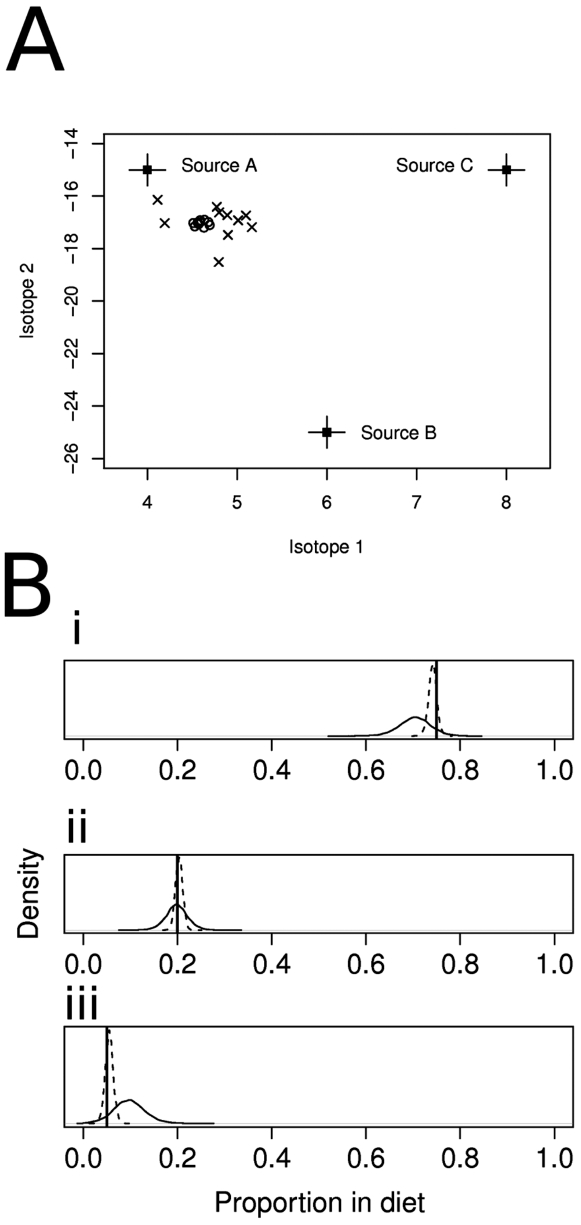
Two example simulated datasets and corresponding model performance on estimating the underlying parameters. (A) Consumer (open circles and crosses) and source (filled squares) isotope values from two data sets with different between-individual variability in the animal isotope measurements (standard deviation *σ* = 0.1 for open circles, compared with 0.5 for crosses). (B) Posterior density estimates of the proportions for both models. The true values of the proportions are shown in the vertical lines. In both cases, the true proportions are inside the 95% credibility intervals of the posterior distributions.

First, we illustrate the model with a simulated example involving 2 unique isotope measurements on 10 organisms whose diets comprise 3 different uncertain sources: A, B and C; in SIAR these are treated as normally distributed. We set TEFs to zero and the concentration dependencies as equal with no loss of generality. Setting the trophic enrichment values to zero mean and zero standard deviation has no bearing on the performance of the model. Adding variation here is mathematically identical to increasing variation on the sources since the variances are combined additively in the formation of the likelihood function: 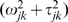 in equation 9 below. The likelihood function is then used to calculate the probability of the data given the model. Conversely, including a non-zero mean TEF simply offsets the data geometrically in isotope-space and has no other bearing on model performance. We simulate data from a known set of true proportions: 0.75, 0.2 and 0.05 for sources A, B and C respectively. Similarly, by varying the residual error parameter 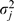 when generating simulated data, we can see how the model responds to differing levels of uncertainty (Fig 1).

### Generating simple test data sets

Below we outline pseudo-code for generating the data sets used for testing the SIAR coverage properties. The user first has to input the number of data sets required for testing (we used 1000), the number of consumers required for each data set (we use 10), as well as lower and upper limits on the number of sources (we use 3 to 5) and the number of isotopes (we use 2 to 3). Key to the pseudo-code is the likelihood function (which applies when the concentration dependence parameters are set equal *q_1_* = *q_2_*… = *q_k_*) based on a normal distribution with a mean and variance:
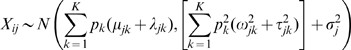
(9)Scenario 1 can then be created via the following pseudo-code:

Loop dataset number;

1. Generate a random set of proportions, *p* from a Dirichlet distribution with all *α_k_* = 1.
2. Generate source means (*μ*) based on a random sample from a normal distribution with mean 0 and standard deviation 10.
3. Generate source standard deviations (*ω*) based on absolute normal distributions (to ensure only positive values) with mean 0 and standard deviation 2.
4. Similarly generate fractionation correction means (*λ*) and standard deviations (*τ*) from normal and absolute normal distributions with means 0 and standard deviations both 1.
5. Generate consumer means (as given by the mean of the likelihood function eq 9) as a proportion-weighted sum of source and correction means
6. Generate residual standard errors (*σ*) as absolute normal distributions with standard deviation 1.
7. Generate consumer standard deviations (right hand side of likelihood function eq 9) as a weighted sum of the squared proportions times the sum of the source and correction variances. Finally add on the residual variance.
8. Generate consumer values (*X*) from the consumer means and standard deviations.
9. Run the SIAR model for 200,000 iterations.
10. Check whether estimated 95% credibility intervals for each proportion contain the original generated proportions.

Repeat for next data set.

**Figure 2:**
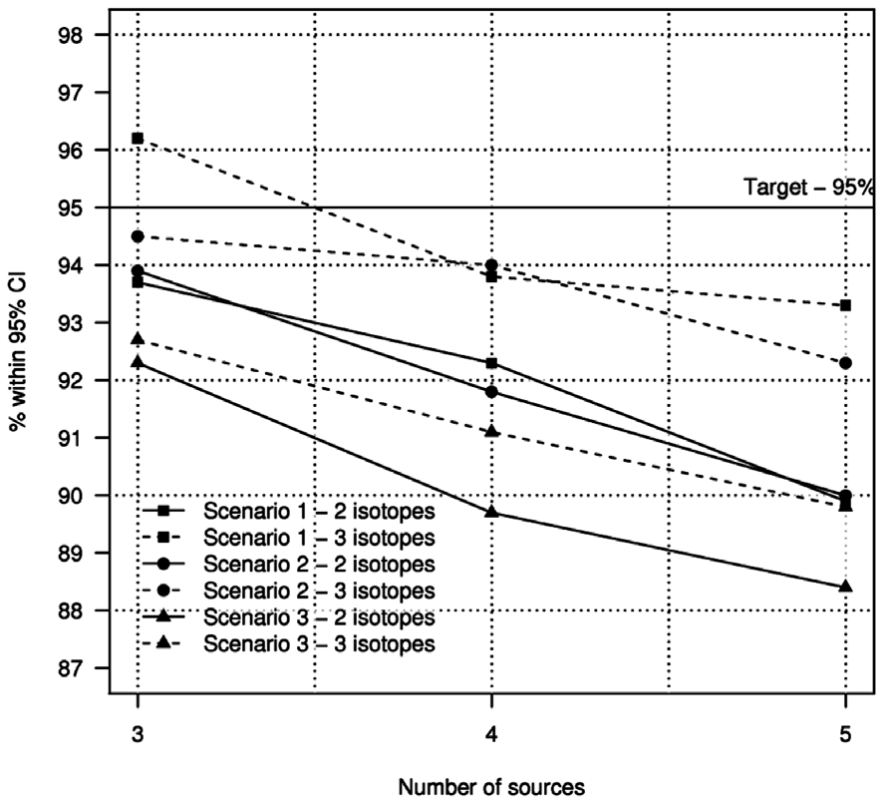
Proportion of 1000 simulated data sets where true values lie inside 95% intervals. The model performs well for all of the different scenarios considered. The figure shows the deterioration of model predictions as the number of sources is increased. Performance can be improved by increasing the number of isotopes used.

The values reported in Figure 2 show the estimated proportion of the 1000 data sets inside the 95% credibility interval.

### More complex data structures

Second, we conduct a fuller examination of the model, picking a selection of ‘reasonable’ scenarios and test how often the simulated true proportions lie inside the 95% credible intervals of the estimates. Clearly, it is impossible to examine all possible scenarios; the 3 we consider are:

1. Model as given, with normally distributed error term *ε_ij_*, as well as normally distributed sources and correction values.
2. Model as given, with *t*_4_-distributed error terms (this is Student's t-distribution with 4 degrees of freedom [21]) on the consumer isotope values, as well as *t*_4_-distributed sources and correction values. The *t*_4_ distribution provides long-tailed errors which may be more natural when source and TEF standard deviations are based on few observations (*N_obs_*≤5).
3. Model as given, but where the two closest sources have been combined to produce a single source.

These more complex scenarios of the sensitivity analysis are easily re-created by adapting the above steps for the simple case by altering the distributions of random variables and averaging across sources when combining the two nearest sources. In each case, 1000 simulated data sets of 10 target organism values were produced for data with between 2 and 3 isotopes and 3 and 5 sources. SIAR performs extremely well (Fig 2), given that some simulations, by chance, represent biological extremes. The model performs increasingly poorly as the number of sources increases (Fig 1). However, increasing the number of isotopes sustains the predictive power of the model as the number of sources increase. The model even performs acceptably in scenarios 2 and 3 where some key model assumptions are violated.

## Discussion

SIAR works exceptionally well for numerous datasets, appearing robust to violations of its core assumptions (Figs 1 & 2). Users can therefore be confident of the estimated dietary composition of consumers, even in underdetermined systems. Further, they can be assured that uncertainty and variation in parameters is included in these estimates, meaning that we now have a tool to investigate complex dietary systems with greater quantitative rigour than before. Additionally, SIAR includes capability for inclusion of concentration dependence which has shown to be an essential consideration in some circumstances [20].

The Bayesian approach naturally propagates sources of uncertainty into posterior probability distributions, and as such we can make statements about which solutions are more likely than others, allowing us to use these estimates in down-stream statistical models such as relating proportion of a particular source to another measured parameter of interest such as fitness. Ideally one would bolt another Bayesian model onto the SIAR output and use the full posterior distribution. However, such techniques are not currently widely available to ecologists. Instead, since the posterior contains information on which parameters are more likely than others, a measure of central tendency (preferably the mode) could be used and passed into standard frequentist generalised linear models, particularly if the posterior distributions of interest are precise and not highly skewed. We caution users to be aware that the posterior dietary proportion estimates may be highly uncertain and that single summary values (such as the modes) should be used with care. There is also no reason to expect the modes of the marginal posterior distributions to sum-to-unity: something that is not an issue if the full posterior distribution is used in down-stream analyses.

Not surprisingly there are caveats to consider before applying SIAR (several that are common to all mixing models). Some of these are:

1. SIAR can produce precise estimates, but the underlying model may remain undetermined and thus the outputs represent probable solutions.
2. SIAR (reasonably) assumes that the variability associated with sources and the uncertainty associated with TEFs is normally distributed. If it is suspected that the distributions depart from this assumption then it is possible to change the likelihood function in SIAR (requiring non-trivial recoding).
3. SIAR currently assumes that no isotopic routing occurs within the body of the consumer and that all isotopes are assimilated equally [22]. We urge researches to satisfy themselves with the validity of this assumption as violation may results in misleading results.
4. SIAR will always attempt to fit a model, even if the sources lie outside of the isotopic mixing polygon [20]. Researchers are urged to examine their data carefully before using any mixing models.

Recent quantitative advances allow comparison of community structure based on isotope data alone – in *δ*-space [23], [24]. However, now that Bayesian approaches can yield robust estimates of diet, the prospect of using diet composition to describe community structure (i.e. in p-space) [25], and quantifying competition arising in overlapping niches is becoming a real possibility.

In most instances it will be the causes or consequences of dietary differences that are of interest to the researcher. The Bayesian approach allows further development via the model output, for example the inclusion of the dietary proportions with their uncertainty in generalised linear models to relate diet with other explanatory variables such as the inclusion of random effects in MixSIR [26]. Furthermore, these mixing models are equally applicable to other mixing problems such as identifying pollution sources. SIAR potentially opens up a host of possibilities for addressing key ecological questions and we envisage it developing continually as open-source software, becoming a holistic resource for performing a range of generic analyses relating to stable isotope techniques.
